# An Ayurvedic Medication (Chyawanprash) as a Prophylaxis for Non-Communicable Disease and Communicable Disease: A Protocol for Systematic Review and Meta-Analysis

**DOI:** 10.7759/cureus.47555

**Published:** 2023-10-24

**Authors:** Shubham Sharma, Khushboo Kumari, Gayathree Sethuraman, Maya M Abdelwahab, Suhasini Sivaperumal Yadav, Vangala Nandini

**Affiliations:** 1 Public Health, Indian Institute of Public Health Gandhinagar, Gandhinagar, IND; 2 Public Health, Indian Council of Medical Research, Rajendra Memorial Research Institute of Medical Sciences, Patna, IND; 3 Public Health, Triyog Ayurveda Hospital, Chennai, IND; 4 Faculty of Medicine, Helwan University, Cairo, EGY; 5 Public Health, Collectives for Integrated Livelihood Initiatives "MADHUBAN", Mumbai, IND; 6 Pharmacy, Annamacharya College of Pharmacy, Rajampeta, IND

**Keywords:** srma, protocol, communicable disease, non communicable disease, chyawanprash

## Abstract

Globally, non-communicable diseases (NCDs) and communicable diseases (CDs) are on the rise, posing a significant public health threat. A holistic ayurvedic preparation called chyawanprash (CP) has shown positive outcomes in NCDs and CDs. Hence, we aimed to report the outcomes in a systematic manner. To determine the safety, efficacy, healthcare utilization, and quality of life of CP as an optional therapy for NCD and CD management. This systematic review will adhere to PRISMA-P and Cochrane guidelines for methodological considerations. It will evaluate CP efficacy in diverse populations, considering Ayurvedic and non-Ayurvedic comparators. The study design will encompass randomized controlled trials (RCTs) published from 2010 to 2023 in healthcare settings, controlled environments, and communities. We will also analyze primary outcomes related to immunity biomarkers, vital signs, and secondary outcomes such as quality of life. Data sources and search strategy will involve systematic searches in databases such as Cochrane, PubMed, Google Scholar, Web of Science, and Scopus using MeSH terms and Boolean operators. Screening and data extraction will follow a standardized form with four independent reviewers. Quality assessment will use the Cochrane risk of bias tool. The systematic review will provide an exhaustive summary of the effectiveness and safety of CP to address the growing burden of NCDs and CDs. Registration: CRD42023418994.

## Introduction and background

In recent years, the global healthcare landscape has witnessed a concerning rise in the prevalence of two distinct yet interconnected types of disease: non-communicable diseases (NCDs) and communicable diseases (CDs). NCDs, often referred to as chronic diseases, encompassed a wide range of conditions, such as cardiovascular diseases, diabetes, cancer, and respiratory disorders. On the other hand, CDs included infectious diseases caused by pathogens like bacteria, viruses, and parasites, with notable examples being malaria, tuberculosis, HIV/AIDS, and the COVID-19 pandemic that swept across the world [1,2]. Globally, communicable diseases (CD) (for example, tuberculosis, malaria, HIV/AIDS, etc.) were the leading cause of death worldwide, but medical advancement, rapid urbanization, sedentary lifestyles, unhealthy diets, and environmental factors have contributed to the increasing burden of mortality and morbidity due to chronic diseases [3]. As a result, CDs, as well as NCDs, are becoming a double-edged sword and a major public health concern.

The infectious disease tuberculosis (TB), for example, has been well known for its association and increased vulnerability in patients with NCDs (diabetes mellitus, smoking, alcoholism, chronic lung diseases, cancer, immunosuppressive treatment, malnutrition), and it has emerged as a critical clinical and public health challenge [4,5]. Recent pandemics have brought into sharp focus the interaction between NCDs and CDs. There had been research showing that a high percentage of hospitalized patients due to COVID-19 had underlying chronic conditions, with hypertension being the most common [6]. The prevalence of NCDs was up to 48% among COVID-19 patients in Wuhan, 71.9% among hospitalized COVID-19 patients in New York City, and 98.9% among deceased COVID-19 patients in Italy [7-9]. Similarly, in India, COVID-19 cases and deaths per million were positively correlated with NCD risk factors such as obesity (0.64, 0.52), hypertension (0.28, 0.16), and diabetes (0.66, 0.46) [10]. A recent study of adults in six nations-China, Ghana, India, Mexico, Russia, and South Africa-found that NCDs were associated with higher out-of-pocket expenses, catastrophic health expenditures (CHE), and more healthcare visits compared to CDs (in Ghana, India, and China) [11]. This should establish the paradigm that the government must implement cost-effective policies to combat CDs and NCDs simultaneously. But the issue remains: Do we truly possess such a miracle cure?

Individuals, society, and civilizations have placed tremendous trust in modern medication and technology, drifting far away from their roots. In Ayurveda, the two words "ayur" (life) and "veda" (knowledge) combine to form the science of nature and healing [12]. Even though the Vedas (Charaka and Sushruta Samhita) date back over 3500 years, Ayurveda still holds principles and operational frameworks that are applicable to solving contemporary medical problems in dynamic and evolving epidemiological contexts [13]. Ayurveda is a personalized and holistic system of medicine with deep logical and philosophical roots, aiming to promote and maintain harmony between the body, mind, and soul. Besides being ethnomedicine, it had been considered a complete medical system that promoted the well-being of mankind on its physical, psychological, philosophical, ethical, and spiritual levels [14]. The practice of Ayurveda is one of the world's most prevalent and culturally rooted traditions of healthcare in India, with codified or uncodified versions of its knowledge being available [15,16]. The Ayurvedic system of medicine employs a variety of modalities, including manual therapies, nutritional therapy, herbal remedies, lifestyle counseling, and yoga exercises based on yoga [17]. Various studies (case reports, randomized controlled trials (RCTs), and meta-analyses) have demonstrated Ayurveda's efficacy and safety as a treatment for various ailments, both communicable and non-communicable [18-20]. It has been demonstrated to be a cost-effective alternative for boosting immunity, with few hazardous side effects and ease of use [21]. The holistic approach of Ayurveda, which addresses the needs of the individual, fills a void in the European health system. As a result, the need for effective natural medicine for patients, coupled with high-quality local healthcare professional training and secure Ayurvedic medications, may become a driving force for Ayurveda throughout Europe [15]. Also, outside of its country of origin, particularly in the US and Europe, professional associations are attempting to set standards for Ayurveda practice and education [15,22].

Rasayana, a sub-discipline of Ayurveda, consisted of several specialized methods for longevity, preventing diseases, halting degenerative processes, and promoting optimal health. Ayurvedic rasayana, Chyawanprash (CP), stood out as one of the most renowned and frequently used Ayurvedic supplements in the Indian subcontinent due to its fewer side-effects and cost-effectiveness compared to conventional synthetic nutraceuticals. It is also referred to as chyavanaprasha, chyavanaprash, chyavanaprasam, and chyawanaprash. CP has stood out as the most significant Rasayana formulation among those described during the classical and medieval eras [23,24]. Since ancient times, chyawanprash has been utilized both as a medicine to improve immunity and lengthen life and as a health supplement due to its many positive health effects. Long before vitamins, minerals, and antioxidant supplements became available, it was one of the foods most valued for its anti-aging properties. The CP formula contains roughly 50 bioactive herbs and herbal extracts (and occasionally more, depending on the brand), which are processed using the traditional Ayurvedic pharmaceutical procedure. The final form of CP has the consistency of jam. The mixture contains several bioactive herbs (with the major ingredient, amla or Indian gooseberry) with broad-spectrum therapeutic potential, including bioactive antioxidants, anti-inflammatory, adaptogenic, cytoprotective, and rejuvenating properties [23]. The efficacy and safety of CP as an immunomodulator have been demonstrated in preclinical trials [25], and its effectiveness in reducing health care resource utilization (HCRU) (ICU use, hospitalization days) and improving quality of life (QoL) (WHO ordinal scale) as an immunomodulator has been demonstrated in high-quality clinical trials [26-28]. However, these interventions and their outcomes presented over different settings and durations have shown mixed results. As immunity plays a vital role in protecting an individual from infectious and non-infectious diseases, CP can play a pivotal role as a prophylaxis in such ailments.

Research work in CP is not yet compiled and analyzed, considering the inconclusive evidence present, heterogeneity in outcomes, and duration of interventions. This motivated us to conduct a systematic review of the efficacy and safety of CP as a prophylaxis for NCDs and CDs. The study will critically review peer-reviewed published literature on only randomized controlled trials (RCTs) to establish their safety and clinical efficacy. This novel review will provide precise insights into CP interventions’ effectiveness in the management of NCDs and CD as a stand-alone or add-on to conventional management.

## Review

Objective

The review has following objectives: i) To evaluate the efficacy and safety of CP as a prophylaxis, either standalone or adjuvant therapy, for NCD and CD management. ii) To evaluate the HCRU QoL of CP as a prophylaxis, either standalone or adjuvant therapy for NCD and CD management.

Materials and methods

This review has followed the preferred reporting items for systematic review and meta-analysis protocols (PRISMA-P) guidelines and the Cochrane Handbook for Systematic Reviews of Interventions for methodological considerations [29,30]. The review did not include any confidential information about participants; hence, no ethical clearance was given for conducting the study. The review has been registered in PROSPERO (CRD42023418994).

Eligibility criteria

Population

Healthy, diseased persons, pregnant and lactating women, aged greater than five years of either sex with/without any pre-existing co-morbid conditions.

Intervention

Ayurvedic preparation chyawanprash (CP), with or without drugs, dosage forms, doses, or scheduled alone or in combination with standard conventional care.

Comparator

Treatments involving Ayurvedic drugs, dosage forms, doses, schedules, non-Ayurvedic interventions, conventional treatments, placebo, waitlist controls, or no treatments.

Outcome

All relevant information with respect to primary and secondary outcomes (subjective and objective) reported in the included studies will be extracted.

Biomarkers are an important indicator of normal biological processes and can be utilized as a tool for clinical outcome measurement [31]. Hence, we have considered biomarkers as primary outcomes. A few examples of biomarkers are reported in Table 1. We will also capture progression-free survival, overall survival, improvement in vital signs, and any adverse events, serious adverse events, or discontinuation by patients due to treatment as a primary outcome.

**Table 1:** Biomarkers for communicable and non-communicable diseases HCRU: Health care resource utilization, QoL: Quality of life

Condition	Biomakers
CDs	Lymphocytes, interleukin-6 (IL-6), interleukin-10 (IL-10), C reactive protein (CRP), interleukin 1 beta (IL-1β), cortisol, tumor necrosis factor alpha (TNF-α) [19]
NCDs	Lipids, triglycerides, lipoprotein (high density lipoprotein (HDL), low density lipoprotein (LDL), triglyceride-rich lipoprotein [TRL]), CRP, creatinine, systolic blood pressure (SBP), diastolic blood pressure (DBP), cholesterol [32]
CD/NCD	CRP, Ferritin, IL-6, tropinon, B-type natriuretic peptide (BNP) [33]

The secondary outcomes will report both subjective and objective measures, which may include HCRU, QoL, severity of disease, and cost.

Study Design

Only a randomized controlled trial (pilot or either phase) with at least two arms and peer-reviewed reporting efficacy, safety, and other parameters (HCRU, QoL, etc.) of CP interventions for the management of NCD and CD, either as a standalone system or adjuvant therapy along with conventional medicine, will be included. Studies in different healthcare settings' controlled environments, as well as those conducted in the community, will be included.

Data Source and Search Strategy

The search for this review was conducted through the following electronic databases: Cochrane, PubMed, Google Scholar, Web of Science, Scopus, and citation search. Only articles published between 2010 and 2023 will be included in the review.

The search strategy will include the MeSH terms and the Boolean operators ‘AND’ and ‘OR’. Chyawanprash OR chyavanprash OR ratnaprash AND human study was used. The search strategy may differ based on the requirements of the respective electronic database. The search strategy for PubMed is shown in Additional File 1.

Screening and analysis

Data Security

All the articles retrieved from the electronic databases will be kept securely in password-protected Excel sheets. Access to the data will only be available to the authors of this review. The study will be conducted using COVIDENCE software (free trial version for LMIC countries) [34].

Study Screening and Selection

The screening of all retrieved articles will be completed in two phases, i.e., Level 1 screening (L1) and Level 2 screening (L2). L1 consists of title and abstract screening, and L2 consists of full text screening. Four independent reviewers will screen the articles based on the inclusion-exclusion criteria. The articles selected at L1 will be retrieved for full text and independently assessed for eligibility by four reviewers. Any disagreement over the inclusion or exclusion of the articles at any level will be resolved by the fifth reviewer. As per the need, additional/missing information or articles related to the clinical trials will be obtained from the corresponding author or principal investigator of the study through e-mail (two emails will be sent). The PICOT (inclusion and exclusion criteria) is reported in Additional File 2.

Data Extraction

For the purpose of evaluating research quality and synthesizing the available evidence, data from the included studies will be extracted using a standard, pre-piloted form. Study setting (including country), study population and participant demographics and baseline characteristics, intervention and comparator used, specifics of the intervention and control conditions, study methodology, recruitment and study completion rates, outcomes and times of measurement, indicators of acceptability to users, and information for assessing the risk of bias are among the data that will be extracted. Data will be independently extracted by four reviewers, and discrepancies will be found and discussed (where required, with a fifth author). The study authors will be emailed to request any missing data.

Quality Assessment

Four reviewers will independently assess the risk of bias for the included randomized controlled trial, as per the recommendation of the Cochrane risk of bias [ROB] tool, reported in Table 2 [30].

**Table 2:** Risk of bias

Levels	Description
Selection bias	Treatment allocation concealment
Performance bias and detection bias	Blinding
Attrition bias	Incomplete outcome data
Reporting bias	Selective reporting
Other bias	Any other potential bias

**Figure 1:**
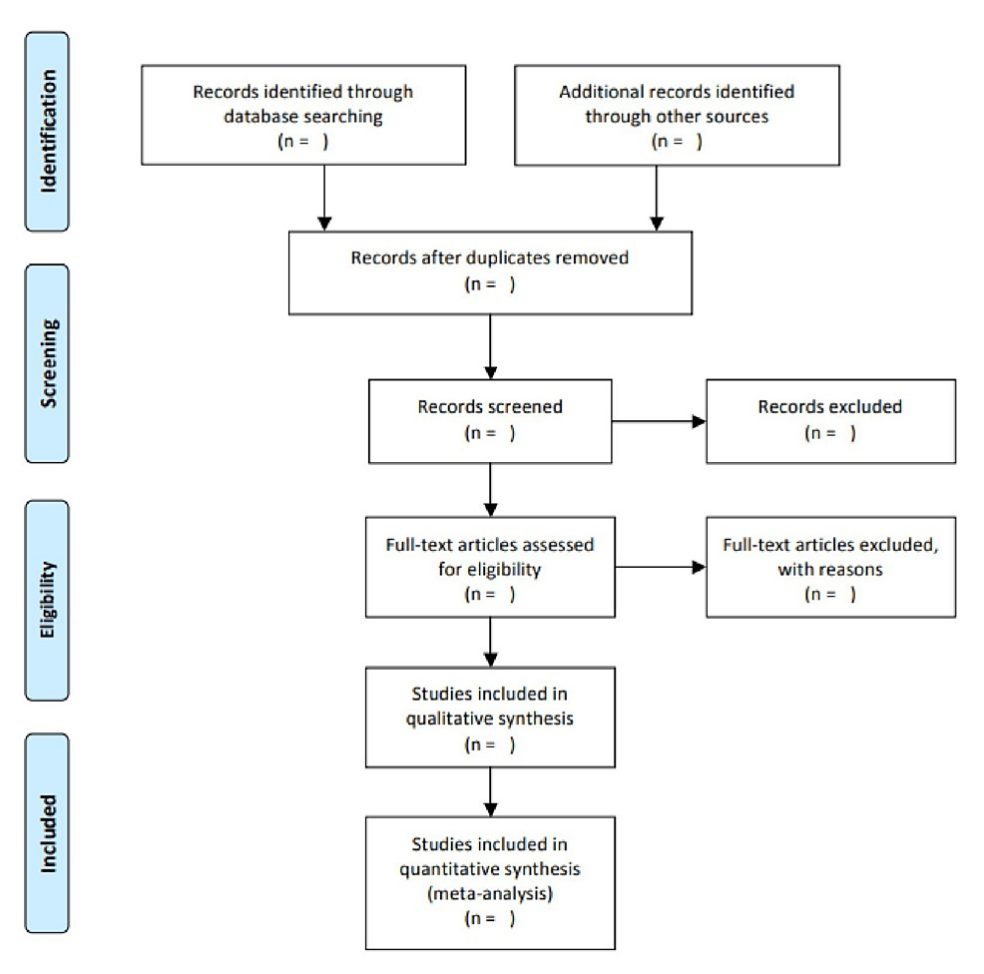
PRISMA flow chart

**Figure 2:**
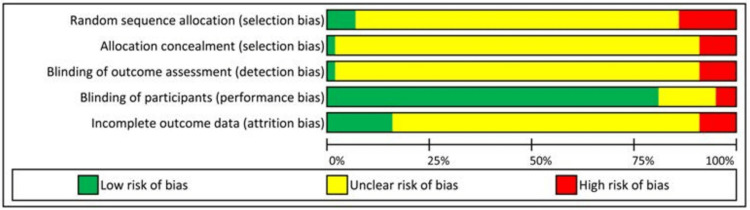
Risk of bias assessment

The responses for each of the five parameters of the ROB tool will be either low risk, moderate risk, or high risk. The pictorial representation of include-excluded articles (PRISMA flowchart) and ROB tool results are represented in Figures 1, 2, respectively.

Data Analysis

All the included articles after L2 screening will be analyzed for descriptive and analytical analysis. The descriptive analysis will be reported in a narrative manner, consisting of: intervention and comparator description, setting of the trial, population characteristics in the form of range, proportions, etc.

For studies in which pooled estimates can be obtained, meta-analysis will be conducted in the respective analytical software to report the pooled effect. Binary outcome data will be combined using relative risk, and for continuous outcomes, data will be summarized by arithmetic means and standard deviation (SD); a 95% confidence interval will be calculated for both outcomes, respectively. Studies reporting more than two arms will be analyzed by pooling the mean and SD from the two experimental groups for the outcome measure. By examining the forest plots for overlapping confidence intervals, using the Chi-square test with a p-value of <0.05 to indicate statistical significance, and using the I2 test with a value of 50% to represent moderate levels of heterogeneity, heterogeneity among trials will be evaluated. A random-effects model will be employed if heterogeneity is found and the combination of studies is still thought to be clinically significant. The sensitivity analysis will be conducted to check the robustness of the results, provided sufficient studies are included in the meta-analysis. We will report the publication bias either visually using a funnel plot or through a funnel plot and Egger’s statistical test based on the number of included articles.

Discussion

In this systematic review protocol, we will evaluate published RCTs that have evaluated chyawanprash's efficacy in communicable and noncommunicable diseases over the past 13 years. In addition to selecting only peer-reviewed published articles in English, this study has the following strengths: It may assist clinicians and patients with prophylaxis against CD and NCD. Clinical research may be conducted based on the outcomes of the systematic review. CP is appealing to some patients because the inconveniences are generally limited compared with other nutraceuticals. In conclusion, this study will be the first to assess the effectiveness of CP for the preventative treatment of CD and NCD. It will offer trustworthy support for its wide-ranging application.

However, this systematic review also has several limitations. The search has not included gray literature or language restrictions (only English articles). Different evaluation standards resulting from different CP dosages may also be responsible for the high heterogeneity.

## Conclusions

This systematic review of ayurvedic interventions in healthy, diseased, pregnant, and lactating women will provide a detailed summary of the evidence for the efficacy and safety of chyawanprash in communicable and non-communicable diseases. In the event of a protocol amendment, we will provide the date, a description of the change, and a rationale for the change.

## Appendices

**Table 3:** PubMed search strategy

Search number	Query	Filters	Search Details	Results	Time
5	#1 OR #2 OR #3	from 2010 to 2023	("chyavanprash"[Supplementa ry Concept] OR "chyavanprash"[All Fields] OR "chyawanprash"[All Fields] OR "chyavanprash"[Supplementar y Concept] OR "chyavanprash"[All Fields] OR "chyavanprash"[All Fields] OR "ratnaprash"[All Fields]) AND (2010:2023[pdat])	27	08:24:35
4	#1 OR #2 OR #3		"chyavanprash"[Supplementar y Concept] OR "chyavanprash"[All Fields] OR "chyawanprash"[All Fields] OR "chyavanprash"[Supplementar y Concept] OR "chyavanprash"[All Fields] OR "chyavanprash"[All Fields] OR "ratnaprash"[All Fields]	30	08:24:28
3	Ratnaprash		"ratnaprash"[All Fields]	1	08:23:34
2	Chyavanpras h		"chyavanprash"[Supplementar y Concept] OR "chyavanprash"[All Fields] OR "chyavanprash"[All Fields]	14	08:23:22
1	Chyawanpras h		"chyavanprash"[Supplementar y Concept] OR "chyavanprash"[All Fields] OR "chyawanprash"[All Fields]	29	08:23:04

**Table 4:** PICOT

Description	Inclusion	Exclusion
Population	Healthy, diseased, children’s (5 to 17 years), adults (>= 18 years), either gender, participants with co-morbid conditions, pregnant and lactating women	Children below 5 years of age. Pre-clinical trial/Animal
Intervention	Chyawanprash (with or without milk, and other natural ingredients/medication)	Except what’s in inclusion criteria
Comparator	Allopathy drugs, COVID-19 vaccine, AYUSH, natural ingredients, standard of care, milk. Any or none.	Except what’s in inclusion criteria
Outcome	Primary outcomes will report progression free survival, overall survival, improvement in vital signs, and any adverse events, serious adverse events, discontinuation by patients due to treatment. The secondary outcomes will report HCRU, QoL, severity of disease, and cost.	No reason to exclude
Study design	RCTs (Pilot, phase (I, II, and III)), at least two arm trial	Observational study, SRMA, single arm RCT, Quasi RCT, qualitative study, pre-clinical trial, review article, pooled analysis, other language, letter to editor, and non-peer reviewed articles. Exclude and highlight all relevant SRMA, IMA, Economic study (if following other variables of PICOT).
Time line and language	January 2010 to February 2023 English language only	Except what’s in inclusion criteria
